# Successful Gastric Volvulus Reduction and Gastropexy Using a Dual Endoscope Technique

**DOI:** 10.1155/2014/136381

**Published:** 2014-01-19

**Authors:** Laith H. Jamil, Brian L. Huang, David C. Kunkel, Vijay Jayaraman, Edy E. Soffer

**Affiliations:** ^1^Division of Digestive Diseases, Cedars-Sinai Medical Center, Los Angeles, CA, USA; ^2^Department of Medicine, Cedars Sinai Medical Center, 8700 Beverly Boulevard, Los Angeles, CA 90048, USA; ^3^Department of Gastroenterology, University of Southern California, Los Angeles, CA, USA

## Abstract

Gastric volvulus is a life threatening condition characterized by an abnormal rotation of the stomach around an axis. Although the first line treatment of this disorder is surgical, we report here a case of gastric volvulus that was endoscopically managed using a novel strategy. An 83-year-old female with a history of pancreatic cancer status postpylorus-preserving Whipple procedure presented with a cecal volvulus requiring right hemicolectomy. Postoperative imaging included a CT scan and upper GI series that showed a gastric volvulus with the antrum located above the diaphragm. An upper endoscopy was advanced through the pylorus into the duodenum and left in this position to keep the stomach under the diaphragm. A second pediatric endoscope was advanced alongside and used to complete percutaneous endoscopic gastrostomy (PEG) placement for anterior gastropexy. The patient's volvulus resolved and there were no complications. From our review of the literature, the dual endoscopic technique employed here has not been previously described. Patients who are poor surgical candidates or those who do not require emergent surgery can possibly benefit the most from similar minimally invasive endoscopic procedures as described here.

## 1. Introduction

Gastric volvulus is a relatively rare condition that is characterized by an abnormal rotation of the stomach around an axis. Rotation of the stomach along the longitudinal axis is termed organoaxial volvulus, while rotation along the transverse axis is termed mesenteroaxial volvulus [1, 2]. Although gastric volvulus can be the primary condition, it is usually secondary to other disorders such as adhesions, diaphragmatic hernias, and paraesophageal hiatal hernias, among other risk factors [2, 3]. It has been shown to occur as a complication of certain surgical procedures as well [4, 5]. This disorder was first described by Berti et al. in 1866 on postmortem examination; further studies have established that patients classically present with epigastric pain, nonproductive retching, and failure to pass a nasogastric tube [1, 6].

Gastric volvulus is potentially lifethreatening with mortality rates as high as 50% as the major causes of death are secondary to complications from strangulation including perforation, hemorrhage, and shock [1, 2, 7]. Presently, first line treatment of this disorder is still with open and more recently with laparoscopic surgery [1, 8]. Although the exact role of endoscopy is still not entirely clear in treating volvulus, endoscopic techniques for volvulus reduction have been successfully employed in high surgical risk patients without signs of ischemia [9–12]. We report here a unique case of gastric volvulus that was endoscopically managed using a novel strategy that to our knowledge has not been previously described in the literature.

## 2. Case Presentation

An 83-year-old female with a history of pancreatic cancer status postpylorus-preserving Whipple procedure presented to an outside hospital with right lower quadrant abdominal pain secondary to cecal volvulus. After transfer to this institution, she developed ischemic bowel that required right hemicolectomy and primary ileocolonic resection.

### 2.1. Endoscopy

**Figure 1:**
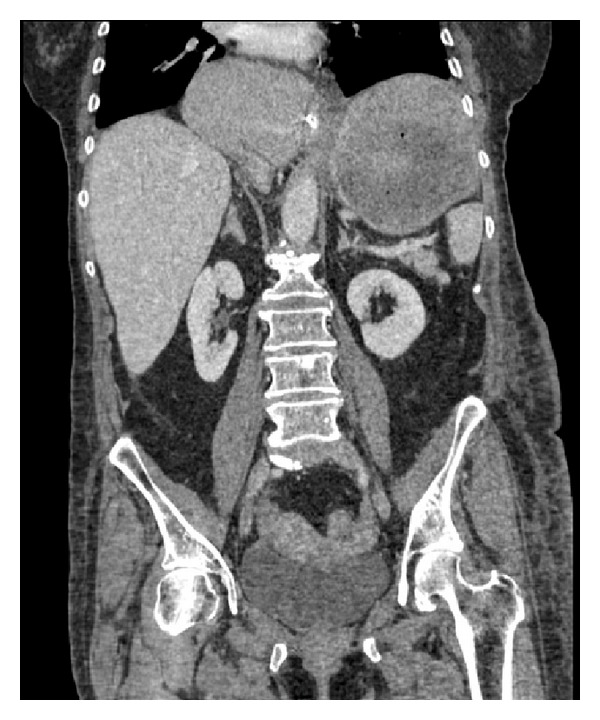
Coronal CT showing herniated stomach above the diaphragm (arrow).

**Figure 2:**
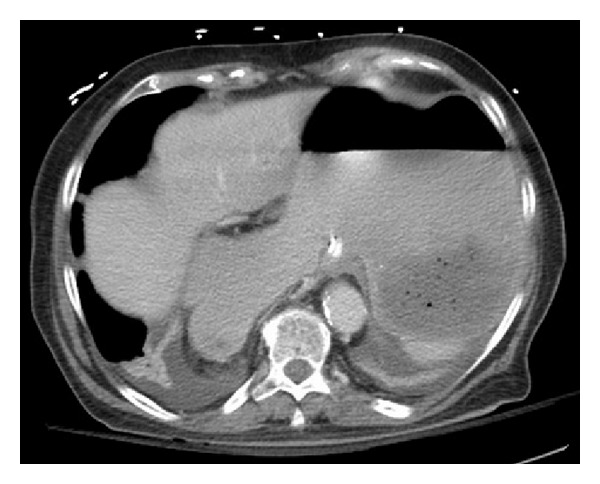
Transverse CT showing severe stomach distension.

**Figure 3:**
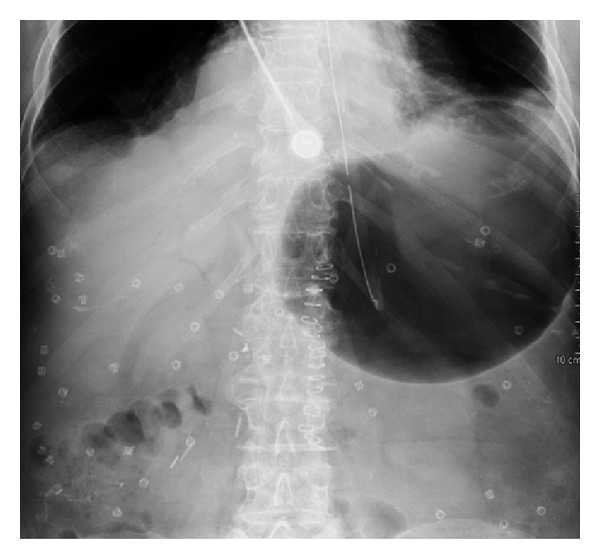
Plain film prior to contrast administration showing stomach distension.

**Figure 4:**
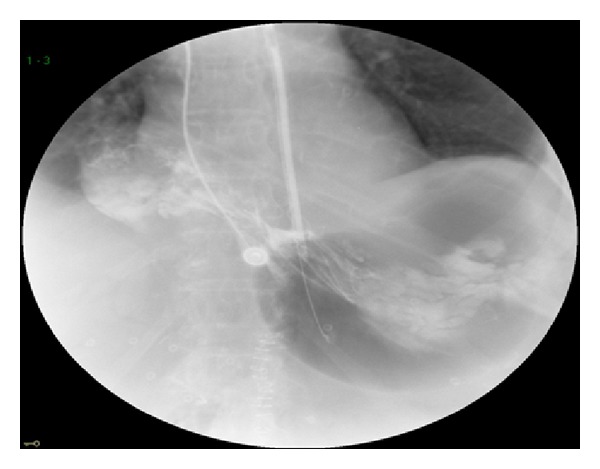
Upper GI series showing NG tube delivering contrast into stomach and supradiaphragmatic antrum. Note that contrast does not flow out of the antrum.

The patient's postoperative course was complicated by abdominal pain with nausea and vomiting. Follow-up imaging including a CT scan (Figures 1 and 2) showed a significantly distended stomach and gastric volvulus with the antrum located above the diaphragm. An upper GI series (Figures 3 and 4) confirmed these findings and she was brought to the endoscopy suite. An upper endoscopy (performed by LHJ) using an adult GIF-H180 revealed a sliding hiatal hernia and a U-shaped stomach. The endoscope had to be retroflexed and advanced adjacent to the gastroesophageal junction to enter the antrum. As the endoscope was advanced through the pylorus into the duodenum, the stomach was noted to assume its normal orientation. Under fluoroscopy, a stiff Jagwire was placed in the duodenum to help maintain this position, but withdrawal of the endoscope caused the antrum to prolapse back into the intrathoracic cavity. At this time, the decision was made to push down the greater curvature of the stomach with the endoscope and straighten out the gastric antrum. This scope was then detached from the processor and left in this position with the tip in the second portion of the duodenum, in the long position, to keep the entire stomach under the diaphragm. A second 4.9 mm pediatric endoscope was advanced alongside the adult endoscope and used to complete percutaneous endoscopic gastrostomy (PEG) placement for anterior gastropexy. We noted that the previously seen prolapse of the gastric antrum through the diaphragmatic defect into the intrathoracic region was no longer seen. The antrum maintained its position and the previously seen U-shaped stomach was less tortuous. Both endoscopes were then withdrawn and there were no complications from this procedure.

### 2.2. Followup

The patient's remaining hospital course was uneventful. She continued to recover, and at time of discharge, her bowel function normalized and she was able to tolerate a full liquid diet. She was seen in clinic two months after discharge without any complications from her PEG tube.

## 3. Discussion

Gastric volvulus is a relatively uncommon condition that can be managed with surgical and endoscopic approaches. Traditional surgical techniques such as gastrojejunostomy, partial gastrectomy, and fundoantral gastrogastrostomy are no longer used due to newer less-invasive procedures [6]. Other techniques have been employed including endoscopic derotation by manipulating the instrument into a “J-shape” and rotating it in a clockwise or counterclockwise manner [13]. While endoscopic derotation has had some documented success, it is at best a temporary solution to such a recurrent condition [14, 15]. As such, recent literature has suggested techniques involving endoscopic anterior gastropexy (used in the current case) as a more permanent treatment to this condition [6].

Treating gastric volvulus can be technically challenging and other studies have utilized dual PEG tube placement and laparoscopic gastropexy to allow for better stomach orientation during the procedure and to decrease relapse rates after the procedure [16, 17]. The technique described in our case report is unique in that, although not as invasive as surgical intervention, it allows the endoscopist to have improved spatial manipulation over the stomach without the need for a second PEG tube or laparoscopic gastropexy. From our review of the current literature, the dual endoscopic technique employed in this case report has not been previously described to treat gastric volvulus. Furthermore, patients who are poor surgical candidates or those who do not require emergent surgical intervention can possibly benefit the most from minimally invasive endoscopic procedures, such as the case detailed here.
